# Supplementary material for: AI-based Hepatic Steatosis Detection and Integrated Hepatic Assessment from Cardiac CT Attenuation Scans Enhances All-cause Mortality Risk Stratification: A Multi-center Study
Source: medRxiv. 2025 Jul 17:2025.06.09.25329157. Originally published 2025 Jun 11. Preprint. [Version 2] doi: 10.1101/2025.06.09.25329157 (PMC12204434; doi:10.1101/2025.06.09.25329157)
Supplement: Supplement 1 [file media-1.pdf]

AI-based Hepatic Steatosis Detection and Integrated Hepatic Assessment from Cardiac CT Attenuation Scans Enhances All-cause Mortality Risk Stratification: A Multi-center Study

Supplementary appendix

Supplementary Methods

We developed and externally validated an eXtreme gradient boosting (XGBoost) decision tree model to combine hepatic measures and three demographic variables which were widely available and modified the association of hepatic steatosis with mortality to derive a liver risk index (LIRI) for integrated liver assessment, and the all-cause mortality status was used as ground truth label for training the model with a supervised binary classification task. Patient cases from one positron emission tomography (PET) site (Brigham and Women's Hospital (BWH)) and one single-photon emission computed tomography (SPECT) site (University of Calgary (CALGARY)) were used as external testing site data for evaluating prognostic value of LIRI while the cases from the other four PET sites (Cedars-Sinai Medical Center (CSMC), Intermountain Healthcare (ITMT), Mayo Clinic (MAYO), and University of Naples Federico II (NAPLES)) and the other three SPECT sites (Yale University (YALE), Columbia University (COLUMBIA), University of Ottawa (OTTAWA)) were used as model development dataset.

Data preprocessing

The 0.5th and 99.5th percentiles of numerical features were calculated over the model development dataset to truncate the features within these bounds to avoid potential outliers. The numerical features were then normalized to fall between 0 and 1. Subsequently, the mean and standard deviation of the numerical features over the model development dataset were computed to standardize the features, ensuring they have a zero mean and unit variance.

External testing scheme

To validate the generalizability of XGBoost models across sites, we trained and validated XGBoost models in an external testing scheme (Supplementary Figure 1).[1] Patients from four PET sites (CSMC, ITMT, MAYO and NAPLES) and three SPECT sites (YALE, COLUMBIA, and OTTAWA) were used as the development dataset to train the XGBoost model, while patients from the remaining PET site (BWH) and the remaining SPECT site (CALGARY) served as the external testing site dataset.

Hyperparameter tuning and selection

The development data was randomly and equally split into five non-overlapping folds. For each of the five folds, it was used as the validation fold, while the remaining four folds were used as the training data to train a model. During hyperparameter tuning (Supplementary Figure 1-A), each candidate hyperparameter configuration was used to train a model on the training data and then evaluated for area under receiver operating characteristic curve (AUC) on the corresponding validation data. The binary logistic loss function was used. The average AUC across all five validation folds was used to select the best-performing hyperparameter configuration. See Supplementary Table 2 for the searching space and the final configuration of the hyperparameter selection.

Model training

During model training (Supplementary Figure 1-B), the development data was randomly split into training (80%) and validation (20%) sets. The training data and the optimally selected hyperparameters were used to train an XGBoost model, while the validation data was used to select the number of trees for prediction on the external testing data. We repeated the model training twice with different random splits of the development data to obtain two models for prediction on the external testing data. In total, two XGBoost models were selected.

Inference and external testing

Both the external testing sites (i.e., BWH and CALGARY) were used for evaluating the prognostic value of LIRI produced by the model. During testing, the two models were used to make predictions for the external testing site data (Supplementary Figure 1-C), with the final prediction being the maximum of the two predictions.

Supplementary Tables

Supplementary Table 1. Overview of key imaging acquisition parameters.

Modality	Site	kVp	Tube current (mAs)	Breathing mode	Spacing (mm)			Dimensionality		
					x	y	z	x	y	z
SPECT/CT	Yale	120	20-150	Normal breathing	0.98	0.98	2.5-5	512	512	25-91
	Calgary	120	20-300	End-expiratory breath hold	0.49-0.98	0.49-0.98	2.5-5	512	512	45-157
	Columbia	90-120	31-125	Normal breathing	1.17	1.17	3	512	512	54-166
	Ottawa	130	24-53	End-expiratory breath hold	0.98	0.98	5	512	512	28-84
PET/CT	CSMC	100	11-13	Normal breathing	1.37	1.37	3	512	512	39-75
	BWH	80-140	10-300	Normal breathing	0.98-1.37	0.98-1.37	2.5-5	512	512	24-79
	Mayo	120-140	20-180	Normal breathing	1.37	1.37	3.75	512	512	27-47
	ITMT	120-130	16-407	Normal breathing	1.37	1.37	2	512	512	82-111
	NAPLES	120-140	35-260	Normal breathing	1.17	1.17	3	512	512	59-60

kVp – kilovoltage peak, SPECT – single-photon emission computed tomography, CT – computed tomography, PET – positron emission tomography, Yale – Yale University, Calgary – University of Calgary, Columbia – Columbia University, Ottawa – University of Ottawa, CSMC – Cedars-Sinai Medical Center, BWH – Brigham and Women's Hospital, Mayo – Mayo Clinic, ITMT – Intermountain Healthcare

Supplementary Table 2. Searching space for hyperparameter tuning and final selected hyperparameters for training eXtreme Gradient Boosting (XGBoost) model.

Hyperparameter	Searching space	Selected value
"**max_depth**": maximum depth of a tree with larger value for more complex model	3, 5, 7	5
"**learning_rate**": step size or scaling coefficient with larger value for more contribution of a tree prediction	0.01, 0.05, 0.3	0.3
"**n_estimators**": number of gradient boost trees to use in an XGBoost model with larger value for more complex model	100, 300, 500	100
"**subsample**": sampling ratio of training instances for training each gradient boost tree with larger value for less biased estimation	0.5, 0.7, 0.9	0.7
"**colsample_bytree**": sampling ratio of features for training each gradient boost tree with larger value for more complex model	0.5, 0.7, 0.9	0.9
"**scale_pos_weight**": scaling coefficient of loss for positive instances with larger value for focusing more on improving prediction performance over positive instances	6, 7, 8	8
"**gamma**": minimum loss reduction required for growing a gradient boost tree depth with larger value for shorter tree and less complex model	0, 0.2, 1	0
"**lambda**": coefficient of L_2 regularization on gradient boost tree weights with larger value for smaller model weights and less complex model	0, 0.1, 1	1
"**alpha**": coefficient of L_1 regularization on gradient boost tree weights with larger value for sparser weights and less complex model	0, 0.1, 1	1

Supplementary Table 3. Baseline characteristics for all participants stratified by perfusion imaging modality (SPECT/CT vs PET/CT).

Characteristics	Overall<br>N = 27039	PET<br>N = 16740	SPECT<br>N = 10299	p-value
**Demographics**				
Age [year]	67 (58, 75)	68 (59, 76)	65 (57, 73)	**<0.0001**
age ≥ 60 years	19333 (72%)	12395 (74%)	6938 (67%)	**<0.0001**
Sex				**<0.0001**
Female	11689 (43%)	7068 (42%)	4621 (45%)	
Male	15350 (57%)	9672 (58%)	5678 (55%)	
BMI [kg/m^2]	29.5 (25.5, 34.7)	29.3 (25.4, 34.9)	29.9 (25.8, 34.6)	**0.0152**
Race				**<0.0001**
American Indian	92 (0.3%)	73 (0.4%)	19 (0.2%)	
Asian	580 (2.1%)	414 (2.5%)	166 (1.6%)	
Black	2648 (9.8%)	1452 (8.7%)	1196 (12%)	
Pacific Islander	149 (0.6%)	129 (0.8%)	20 (0.2%)	
White	17186 (64%)	13681 (82%)	3505 (34%)	
Unknown	6384 (24%)	991 (5.9%)	5393 (52%)	
**Medical Conditions**				
Hypertension	20096 (74%)	13732 (82%)	6364 (62%)	**<0.0001**
Diabetes mellitus	8950 (33%)	6072 (36%)	2878 (28%)	**<0.0001**
Dyslipidemia	17645 (65%)	12434 (74%)	5211 (51%)	**<0.0001**
Family history of coronary artery disease	8030 (30%)	5502 (33%)	2528 (25%)	**<0.0001**
Smoking	4644 (17%)	3155 (19%)	1489 (14%)	**<0.0001**
BMI ≥ 30 kg/m^2	12889 (48%)	7775 (46%)	5114 (50%)	**<0.0001**
Stress TPD ≥ 5%	12341 (46%)	8325 (50%)	4016 (39%)	**<0.0001**
LVEF < 50%	4864 (18%)	3212 (19%)	1652 (16%)	**<0.0001**
MFR < 2*	6055 (36%)	6055 (36%)	0 (NA%)	**<0.0001**
**Imaging Measures**				
CAC score [AU]#	2.08 (0.00, 2.95)	2.21 (0.00, 3.01)	1.80 (0.00, 2.84)	**<0.0001**
Stress TPD [%]	4 (2, 10)	5 (2, 12)	3 (1, 8)	**<0.0001**
LVEF [%]	66 (55, 75)	68 (55, 76)	64 (55, 72)	**<0.0001**
MFR*	2.26 (1.77, 2.82)	2.26 (1.77, 2.82)	NA (NA, NA)	
**Hepatic Measures**				
Liver attenuation [HU]	48 (40, 54)	47 (40, 53)	50 (42, 56)	**<0.0001**
Liver SD [HU]	44 (31, 56)	40 (25, 56)	47 (39, 56)	**<0.0001**
Liver entropy [bit]	7.49 (6.97, 7.85)	7.35 (6.67, 7.83)	7.60 (7.32, 7.86)	**<0.0001**
Liver CoV	0.89 (0.61, 1.30)	0.85 (0.53, 1.33)	0.94 (0.73, 1.27)	**<0.0001**
LmS attenuation [HU]	13 (7, 18)	14 (8, 19)	11 (5, 17)	**<0.0001**
LmS SD [HU]	-2.3 (-5.3, 0.0)	-2.2 (-4.3, -0.2)	-2.9 (-8.1, 0.5)	**<0.0001**
LmS entropy [bit]	-0.08 (-0.17, 0.00)	-0.08 (-0.15, -0.01)	-0.08 (-0.22, 0.02)	**<0.0001**
LmS CoV	-0.30 (-0.62, -0.14)	-0.32 (-0.67, -0.15)	-0.27 (-0.54, -0.12)	**<0.0001**
Hepatic steatosis	6579 (24%)	4357 (26%)	2222 (22%)	**<0.0001**
**Outcome**				
Death	4836 (18%)	4200 (25%)	636 (6.2%)	**<0.0001**
Death follow up [year]	3.58 (1.86, 5.15)	4.57 (2.59, 5.73)	2.32 (1.42, 3.65)	**<0.0001**

Values are presented as N (%) or median (IQ1, IQ3). Bold indicates statistical significance with p<0.05. Abnormal stress TPD was defined as stress TPD ≥ 5% and abnormal LVEF was defined as LVEF ≥ 50%. * only PET/CT patients had MFR measurements. # logarithmic transformed CAC score via log10(CAC score+1).

SPECT/CT – single-photon emission computed tomography/computed tomography, PET/CT – positron emission tomography/computed tomography, BMI – body mass index, CAD – coronary artery disease, TPD – total perfusion deficit, LVEF – left ventricular ejection fraction, CAC – coronary artery calcium, AU – Agatston unit, HU – Hounsfield unit, CoV – coefficient of variation, LmS attenuation – difference of liver attenuation minus spleen attenuation, LmS CoV – difference of liver CoV minus

spleen CoV, LmS SD – difference of liver SD minus spleen SD, LmS entropy – difference of liver entropy minus spleen entropy, MFR – myocardial flow reserve

Supplementary Table 4. Hepatic measures for all participants stratified by different characteristics.

Stratified by sex				
Measures	Overall<br>N = 27039	Female<br>N = 11689	Male<br>N = 15350	p-value
Liver attenuation [HU]	48 (40, 54)	50 (42, 56)	47 (39, 53)	**<0.0001**
Liver CoV	0.89 (0.61, 1.30)	0.78 (0.55, 1.11)	0.99 (0.68, 1.43)	**<0.0001**
Liver entropy [bit]	7.49 (6.97, 7.85)	7.34 (6.86, 7.70)	7.60 (7.07, 7.94)	**<0.0001**
Liver SD [HU]	44 (31, 56)	40 (29, 51)	47 (33, 60)	**<0.0001**
LmS attenuation [HU]	13 (7, 18)	13 (8, 19)	12 (6, 18)	**<0.0001**
LmS CoV	-0.30 (-0.62, -0.14)	-0.29 (-0.54, -0.15)	-0.31 (-0.69, -0.13)	**<0.0001**
LmS entropy [bit]	-0.08 (-0.17, 0.00)	-0.09 (-0.18, -0.01)	-0.07 (-0.16, 0.01)	**<0.0001**
LmS SD [HU]	-2.3 (-5.3, 0.0)	-2.5 (-5.2, -0.4)	-2.2 (-5.6, 0.3)	**<0.0001**
Hepatic steatosis	6579 (24%)	2402 (21%)	4177 (27%)	**<0.0001**
Death	4836 (18%)	1900 (16%)	2936 (19%)	**<0.0001**
Death follow up [year]	3.58 (1.86, 5.15)	3.65 (1.98, 5.18)	3.53 (1.79, 5.11)	**<0.0001**
Stratified by BMI				
Measures	Overall<br>N = 27039	BMI < 30 kg/m^2<br>N = 14150	BMI ≥ 30 kg/m^2<br>N = 12889	p-value
Liver attenuation [HU]	48 (40, 54)	52 (46, 57)	44 (34, 50)	**<0.0001**
Liver CoV	0.89 (0.61, 1.30)	0.78 (0.55, 1.07)	1.07 (0.73, 1.59)	**<0.0001**
Liver entropy [bit]	7.49 (6.97, 7.85)	7.37 (6.90, 7.74)	7.62 (7.09, 7.93)	**<0.0001**
Liver SD [HU]	44 (31, 56)	41 (29, 52)	48 (33, 59)	**<0.0001**
LmS attenuation [HU]	13 (7, 18)	13 (8, 18)	12 (4, 18)	**<0.0001**
LmS CoV	-0.30 (-0.62, -0.14)	-0.30 (-0.56, -0.16)	-0.31 (-0.69, -0.10)	**0.0381**
LmS entropy [bit]	-0.08 (-0.17, 0.00)	-0.10 (-0.18, -0.02)	-0.05 (-0.15, 0.03)	**<0.0001**
LmS SD [HU]	-2.3 (-5.3, 0.0)	-2.8 (-5.7, -0.6)	-1.8 (-4.8, 0.7)	**<0.0001**
Hepatic steatosis	6579 (24%)	1586 (11%)	4993 (39%)	**<0.0001**
Death	4836 (18%)	2992 (21%)	1844 (14%)	**<0.0001**
Death follow up [year]	3.58 (1.86, 5.15)	3.54 (1.77, 5.13)	3.63 (1.97, 5.17)	**<0.0001**
Stratified by age				
Measures	Overall<br>N = 27039	< 60 years old<br>N = 7706	≥ 60 years old<br>N = 19333	p-value
Liver attenuation [HU]	48 (40, 54)	48 (37, 55)	49 (41, 54)	**<0.0001**
Liver CoV	0.89 (0.61, 1.30)	0.91 (0.60, 1.36)	0.89 (0.61, 1.28)	**0.0012**
Liver entropy [bit]	7.49 (6.97, 7.85)	7.50 (6.96, 7.84)	7.49 (6.97, 7.85)	0.3798
Liver SD [HU]	44 (31, 56)	44 (30, 56)	44 (31, 56)	0.2198
LmS attenuation [HU]	13 (7, 18)	11 (4, 17)	13 (8, 19)	**<0.0001**
LmS CoV	-0.30 (-0.62, -0.14)	-0.26 (-0.53, -0.10)	-0.32 (-0.65, -0.16)	**<0.0001**
LmS entropy [bit]	-0.08 (-0.17, 0.00)	-0.08 (-0.17, 0.01)	-0.08 (-0.17, 0.00)	0.0977
LmS SD [HU]	-2.3 (-5.3, 0.0)	-2.4 (-5.4, 0.0)	-2.3 (-5.3, 0.0)	0.1847
Hepatic steatosis	6579 (24%)	2342 (30%)	4237 (22%)	**<0.0001**

Death	4836 (18%)	723 (9.4%)	4113 (21%)	**<0.0001**
Death follow up [year]	3.58 (1.86, 5.15)	3.67 (1.82, 5.25)	3.56 (1.88, 5.11)	**0.0341**
Stratified by race				
Measures	Overall<br>N = 19834	Black<br>N = 2648	White<br>N = 17186	p-value
Liver attenuation [HU]	48 (40, 54)	50 (42, 56)	48 (40, 53)	**<0.0001**
Liver CoV	0.92 (0.59, 1.36)	0.94 (0.63, 1.40)	0.92 (0.59, 1.36)	**0.002**
Liver entropy [bit]	7.51 (6.84, 7.92)	7.61 (7.04, 8.01)	7.48 (6.80, 7.90)	**<0.0001**
Liver SD [HU]	44 (28, 59)	48 (32, 63)	44 (28, 58)	**<0.0001**
LmS attenuation [HU]	13 (7, 19)	15 (9, 21)	13 (7, 18)	**<0.0001**
LmS CoV	-0.33 (-0.70, -0.15)	-0.38 (-0.93, -0.17)	-0.33 (-0.67, -0.15)	**<0.0001**
LmS entropy [bit]	-0.08 (-0.17, 0.00)	-0.10 (-0.21, 0.00)	-0.08 (-0.17, 0.00)	**<0.0001**
LmS SD [HU]	-2.4 (-5.5, -0.2)	-3.3 (-8.4, -0.2)	-2.3 (-5.2, -0.2)	**<0.0001**
Hepatic steatosis	4999 (25%)	538 (20%)	4461 (26%)	**<0.0001**
Death	4084 (21%)	560 (21%)	3524 (21%)	0.4463
Death follow up [year]	4.02 (2.00, 5.32)	3.55 (1.73, 6.19)	4.12 (2.03, 5.27)	0.1671
Stratified by diabetes				
Measures	Overall<br>N = 27039	Non-diabetic<br>N = 18089	Diabetic<br>N = 8950	p-value
Liver attenuation [HU]	48 (40, 54)	50 (42, 55)	45 (37, 52)	**<0.0001**
Liver CoV	0.89 (0.61, 1.30)	0.87 (0.60, 1.24)	0.96 (0.63, 1.45)	**<0.0001**
Liver entropy [bit]	7.49 (6.97, 7.85)	7.48 (7.00, 7.82)	7.52 (6.86, 7.89)	**0.0487**
Liver SD [HU]	44 (31, 56)	44 (31, 55)	45 (28, 58)	0.066
LmS attenuation [HU]	13 (7, 18)	13 (7, 18)	12 (5, 18)	**<0.0001**
LmS CoV	-0.30 (-0.62, -0.14)	-0.30 (-0.60, -0.15)	-0.30 (-0.66, -0.11)	**0.0157**
LmS entropy [bit]	-0.08 (-0.17, 0.00)	-0.08 (-0.17, 0.00)	-0.07 (-0.16, 0.01)	**<0.0001**
LmS SD [HU]	-2.3 (-5.3, 0.0)	-2.4 (-5.5, -0.1)	-2.1 (-4.9, 0.3)	**<0.0001**
Hepatic steatosis	6579 (24%)	3658 (20%)	2921 (33%)	**<0.0001**
Death	4836 (18%)	2771 (15%)	2065 (23%)	**<0.0001**
Death follow up [year]	3.58 (1.86, 5.15)	3.60 (1.88, 5.11)	3.56 (1.84, 5.21)	0.9971
Stratified by perfusion				
Measures	Overall<br>N = 27039	Stress TPD < 5%<br>N = 14698	Stress TPD ≥ 5%<br>N = 12341	p-value
Liver attenuation [HU]	48 (40, 54)	49 (41, 55)	47 (40, 53)	**<0.0001**
Liver CoV	0.89 (0.61, 1.30)	0.93 (0.67, 1.33)	0.85 (0.55, 1.26)	**<0.0001**
Liver entropy [bit]	7.49 (6.97, 7.85)	7.56 (7.14, 7.89)	7.38 (6.72, 7.78)	**<0.0001**
Liver SD [HU]	44 (31, 56)	46 (35, 58)	41 (26, 54)	**<0.0001**
LmS attenuation [HU]	13 (7, 18)	13 (7, 18)	12 (6, 18)	**0.004**
LmS CoV	-0.30 (-0.62, -0.14)	-0.33 (-0.67, -0.16)	-0.27 (-0.55, -0.12)	**<0.0001**
LmS entropy [bit]	-0.08 (-0.17, 0.00)	-0.09 (-0.18, -0.01)	-0.06 (-0.15, 0.02)	**<0.0001**
LmS SD [HU]	-2.3 (-5.3, 0.0)	-2.9 (-6.3, -0.4)	-1.7 (-4.3, 0.4)	**<0.0001**
Hepatic steatosis	6579 (24%)	3393 (23%)	3186 (26%)	**<0.0001**
Death	4836 (18%)	1828 (12%)	3008 (24%)	**<0.0001**

Death follow up [year]	3.58 (1.86, 5.15)	3.67 (1.94, 5.11)	3.46 (1.78, 5.20)	**0.0011**
Stratified by myocardial flow reserve				
Measures	Overall<br>N = 16740	Non-CMD (MFR ≥ 2)<br>N = 10685	CMD (MFR < 2)<br>N = 6055	p-value
Liver attenuation [HU]	47 (40, 53)	48 (40, 53)	47 (39, 53)	**0.0001**
Liver CoV	0.85 (0.53, 1.33)	0.89 (0.55, 1.36)	0.78 (0.50, 1.26)	**<0.0001**
Liver entropy [bit]	7.35 (6.67, 7.83)	7.43 (6.75, 7.86)	7.19 (6.59, 7.79)	**<0.0001**
Liver SD [HU]	40 (25, 56)	42 (27, 56)	36 (24, 54)	**<0.0001**
LmS attenuation [HU]	14 (8, 19)	14 (8, 19)	14 (8, 19)	**0.0096**
LmS CoV	-0.32 (-0.67, -0.15)	-0.33 (-0.66, -0.15)	-0.32 (-0.69, -0.14)	0.4169
LmS entropy [bit]	-0.08 (-0.15, -0.01)	-0.08 (-0.14, -0.01)	-0.08 (-0.16, 0.00)	0.4042
LmS SD [HU]	-2.2 (-4.3, -0.2)	-2.3 (-4.4, -0.4)	-2.0 (-4.2, 0.0)	**<0.0001**
Hepatic steatosis	4357 (26%)	2751 (26%)	1606 (27%)	0.2709
Death	4200 (25%)	1776 (17%)	2424 (40%)	**<0.0001**
Death follow up [year]	4.6 (2.6, 5.7)	4.7 (3.1, 5.8)	4.1 (1.9, 5.7)	**<0.0001**

Values are presented as N (%) or median (IQ1, IQ3)
BMI – body mass index, CAD – coronary artery disease, TPD – total perfusion deficit, LVEF – left ventricular ejection fraction, CAC – coronary artery calcium, CoV – coefficient of variation, LmS attenuation – difference of liver attenuation minus spleen attenuation, LmS CoV – difference of liver CoV minus spleen CoV, LmS standard deviation – difference of liver standard deviation minus spleen standard deviation, LmS entropy – difference of liver entropy minus spleen entropy, MFR – myocardial flow reserve, CMD – coronary microvascular disease, HU – Hounsfield unit

Supplementary Table 5. Maximally selected rank statistics-based optimal cutoffs for our proposed liver and spleen measures in entire population for all-cause mortality prediction in MPI cohort of 27039 patients. For the liver attenuation and the difference of liver attenuation minus spleen attenuation (LmS attenuation), we used the cutoff 40 HU and -10 HU from existing literatures, respectively.[2, 3] We used the maximally selected rank statistic-based cutoff for other hepatic measures.[4]

	Cutoff	Concordance-index [95% CI]
Liver attenuation [HU]	40	0.50 [0.49, 0.51]
Liver CoV	0.5752	0.53 [0.52, 0.53]
LmS attenuation [HU]	-10	0.52 [0.48, 0.53]
LmS CoV	0.0136	0.50 [0.49, 0.51]
LmS entropy [bit]	0.0449	0.52 [0.51, 0.53]
LmS SD [HU]	0.2678	0.48 [0.47, 0.53]

CI – confidence interval, HU – Hounsfield unit, CoV – coefficient of variation, LmS – liver metric minus spleen metric, SD – standard deviation

Supplementary Table 6. Comparison between adjusted hazard ratios of hepatic measure in males and females

In different sex populations		
Factor	Hazard ratio (HR) [95% CI], p value*	
	Female<br>N=11689, 1900 events	Male<br>N=15350, 2936 events
HSIC-based hepatic steatosis	1.02 [0.89, 1.18], 0.7459	**1.21 [1.1, 1.34], 0.0001**
High liver CoV	**0.87 [0.78, 0.97], 0.0093**	**0.8 [0.73, 0.88], <0.0001**
High LmS CoV	1.08 [0.9, 1.31], 0.3996	1.05 [0.91, 1.2], 0.5199
High LmS entropy	1.14 [0.94, 1.38], 0.1895	**1.21 [1.05, 1.39], 0.0096**
High LmS SD	**1.28 [1.08, 1.5], 0.0038**	**1.19 [1.05, 1.36], 0.0059**
In different age populations		
Factor	Hazard ratio (HR) [95% CI], p value*	
	age < 60<br>N=7706, 723 events	age ≥ 60<br>N=19333, 4113 events
HSIC-based hepatic steatosis	0.98 [0.79, 1.2], 0.8216	**1.18 [1.08, 1.28], 0.0003**
High liver CoV	1.01 [0.85, 1.21], 0.9009	**0.81 [0.75, 0.87], <0.0001**
High LmS CoV	1.04 [0.81, 1.33], 0.753	1.08 [0.96, 1.22], 0.2013
High LmS entropy	1.07 [0.81, 1.42], 0.6141	**1.2 [1.06, 1.36], 0.0051**
High LmS SD	**1.72 [1.34, 2.2], <0.0001**	**1.15 [1.03, 1.28], 0.0126**
In different BMI populations		
Factor	Hazard ratio (HR) [95% CI], p value*	
	BMI ≥ 30 kg/m^2<br>N=14150, 2992 events	BMI < 30 kg/m^2<br>N=12889, 1844 events
HSIC-based hepatic steatosis	1.01 [0.9, 1.13], 0.8369	**1.21 [1.07, 1.36], 0.0015**
High liver CoV	0.91 [0.8, 1.02], 0.1051	**0.86 [0.79, 0.93], 0.0004**
High LmS CoV	1.04 [0.9, 1.2], 0.5909	1.1 [0.93, 1.3], 0.2603
High LmS entropy	1.03 [0.87, 1.23], 0.7337	**1.3 [1.11, 1.51], 0.0009**
High LmS SD	**1.23 [1.05, 1.44], 0.0115**	**1.19 [1.05, 1.36], 0.007**
In different race populations		
Factor	Hazard ratio (HR) [95% CI], p value*	
	Black<br>N=2648, 560 events	White<br>N=17186, 3524 events
HSIC-based hepatic steatosis	0.91 [0.7, 1.17], 0.4497	**1.21 [1.11, 1.33], <0.0001**
High liver CoV	0.88 [0.72, 1.08], 0.2172	**0.81 [0.75, 0.88], <0.0001**
High LmS CoV	1.22 [0.88, 1.7], 0.2346	1.1 [0.97, 1.25], 0.1288
High LmS entropy	1.04 [0.72, 1.49], 0.8391	**1.24 [1.08, 1.41], 0.0018**
High LmS SD	1.21 [0.88, 1.66], 0.2502	**1.21 [1.08, 1.36], 0.0012**
In patients with different diabetes status		
Factor	Hazard ratio (HR) [95% CI], p value*	
	With diabetes<br>N=18089, 2771 events	No diabetes<br>N=8950, 2065 events
HSIC-based hepatic steatosis	0.97 [0.86, 1.09], 0.6011	**1.31 [1.17, 1.46], <0.0001**
High liver CoV	**0.87 [0.78, 0.96], 0.0079**	**0.82 [0.75, 0.9], <0.0001**
High LmS CoV	1.06 [0.91, 1.24], 0.4479	1.06 [0.91, 1.24], 0.429

High LmS entropy	**1.25 [1.05, 1.48], 0.0123**	1.14 [0.98, 1.33], 0.0959
High LmS SD	**1.24 [1.06, 1.45], 0.0059**	**1.19 [1.04, 1.35], 0.0119**
In different perfusion populations		
Factor	Hazard ratio (HR) [95% CI], p value*	
	Normal perfusion (stress TPD < 5%) N=14698, 1828 events	Abnormal perfusion (stress TPD ≥ 5%) N=12341, 3008 events
HSIC-based hepatic steatosis	1.11 [0.97, 1.27], 0.1186	**1.15 [1.04, 1.28], 0.0062**
High liver CoV	**0.8 [0.71, 0.9], 0.0002**	**0.87 [0.8, 0.94], 0.0009**
High LmS CoV	0.91 [0.75, 1.11], 0.3524	**1.14 [1, 1.31], 0.0455**
High LmS entropy	**1.25 [1.04, 1.51], 0.0191**	1.15 [1, 1.33], 0.0576
High LmS SD	**1.29 [1.1, 1.51], 0.002**	**1.18 [1.03, 1.34], 0.0138**
In different myocardial flow reserve populations (in PET population only)		
Factor	Adjusted HR [95%], p-value*	
	MFR < 2 N=10685, 1776 events	MFR ≥ 2 N=6055, 2424 events
HSIC-based hepatic steatosis	1.04 [0.93, 1.17], 0.448	1.14 [1, 1.3], 0.0535
High liver CoV	**0.87 [0.79, 0.95], 0.0028**	**0.82 [0.73, 0.91], 0.0004**
High LmS CoV	**1.18 [1.01, 1.37], 0.0383**	1.08 [0.9, 1.28], 0.4178
High LmS entropy	**1.25 [1.06, 1.46], 0.0064**	1.14 [0.94, 1.38], 0.1804
High LmS SD	**1.24 [1.07, 1.42], 0.003**	**1.31 [1.11, 1.54], 0.0015**

Bold indicates statistical significance.

Clinical and perfusion factors: modality, age, body mass index, hypertension, diabetes mellitus, dyslipidemia, family history of coronary artery disease, smoking, stress total perfusion deficit, left ventricle ejection fraction, and deep-learning-coronary artery calcium score. *Adjusted for clinical, perfusion factors and all the other hepatic quantifications. HR of age/BMI/log10(CAC score+1) was per unit increase. For modality, SPECT was the reference modality. LVEF ≥ 50%/Stress TPD<5%/low liver CoV/low LmS CoV/low LmS entropy/low LmS SD was reference group. For diabetes mellitus, dyslipidemia, family history, hypertension, and smoking, patients without them were the reference groups.

CI – confidence interval, BMI – body mass index, CAD – coronary artery disease, TPD – total perfusion deficit, LVEF – left ventricular ejection fraction, CAC – coronary artery calcium, CoV – coefficient of variation, LmS CoV – difference of liver CoV minus spleen CoV, LmS SD – difference of liver SD minus spleen SD, LmS entropy – difference of liver entropy minus spleen entropy, SPECT – single-photon emission computed tomography, PET – positron emission tomography, HSIC – hepatic steatosis imaging criterion

Supplementary Table 7. Characteristics in external testing data of 7200 patients stratified by liver risk index (LIRI) categorized by a cutoff 0.63 which is determined by a maximally selected rank test.[4]

Characteristics	Overall<br>N = 7200	High-risk (> 0.63)<br>N = 1537	Low-risk (≤ 0.63)<br>N = 5663	p-value
**Hepatic measures**				
Liver attenuation	52 (42, 58)	50 (43, 57)	52 (42, 59)	**0.0233**
Liver standard deviation	30 (25, 44)	29 (25, 40)	31 (25, 45)	**<0.0001**
Liver entropy	6.96 (6.62, 7.50)	6.86 (6.63, 7.36)	6.99 (6.62, 7.52)	**<0.0001**
Liver coefficient of variation	0.61 (0.47, 0.87)	0.59 (0.48, 0.82)	0.61 (0.47, 0.89)	**0.0097**
LmS attenuation	12 (6, 18)	14 (8, 21)	12 (6, 17)	**<0.0001**
LmS standard deviation	-2.3 (-4.4, -0.3)	-1.7 (-3.5, 0.3)	-2.5 (-4.6, -0.5)	**<0.0001**
LmS entropy	-0.10 (-0.18, -0.01)	-0.07 (-0.16, 0.01)	-0.11 (-0.19, -0.02)	**<0.0001**
LmS coefficient of variation	-0.22 (-0.38, -0.11)	-0.22 (-0.44, -0.11)	-0.22 (-0.37, -0.11)	0.1309
**Demographics**				
Age	66 (58, 74)	80 (75, 84)	63 (56, 70)	**<0.0001**
Sex				**0.0001**
Female	3295 (46%)	636 (41%)	2659 (47%)	
Male	3905 (54%)	901 (59%)	3004 (53%)	
Body mass index	29.0 (25.3, 33.8)	26.8 (23.0, 31.0)	29.7 (25.9, 34.5)	**<0.0001**
**Outcomes**				
Death	2059 (29%)	775 (50%)	1284 (23%)	**<0.0001**
Death follow up	3.9 (2.0, 6.4)	3.6 (1.7, 5.5)	4.0 (2.1, 6.8)	**<0.0001**

Median (IQR) or frequency (%). Bold indicates statistical significance. * CAC score is logarithmically transformed by log10(CAC score+1). LmS – liver minus spleen. LmS – difference of liver metric minus spleen metric

Supplementary Table 8. Confusion matrices between hepatic steatosis (HS) and clinical/perfusion imaging variables.

	Age	
	< 60 years old	≥ 60 years old
HS	2342 (0.36)	4237 (0.64)
No HS	5364 (0.26)	**15096 (0.74)**

	Sex	
	Male	Female
HS	**4177 (0.63)**	2402 (0.37)
No HS	11173 (0.55)	9287 (0.45)

	Obesity	
	Yes	No
HS	**4993 (0.76)**	1586 (0.24)
No HS	7896 (0.39)	**12564 (0.61)**

	Hypertension	
	Yes	No
HS	**5178 (0.79)**	1401 (0.21)
No HS	14918 (0.73)	5542 (0.27)

	Diabetes	
	Yes	No
HS	2921 (0.44)	3658 (0.56)
No HS	6029 (0.29)	**14431 (0.71)**

	CAC score	
	Non-zero	Zero
HS	**4281 (0.65)**	2298 (0.35)
No HS	14408 (0.70)	6052 (0.30)

	Dyslipidemia	
	Yes	No
HS	**4348 (0.66)**	2231 (0.34)
No HS	13297 (0.65)	7163 (0.35)

	Family history of CAD	
	Yes	No
HS	1889 (0.29)	4690 (0.71)
No HS	6141 (0.30)	**14319 (0.70)**

	Smoking	
	Yes	No
HS	1102 (0.17)	5477 (0.83)
No HS	3542 (0.17)	**16918 (0.83)**

	Stress TPD	
	≥ 5%	< 5%
HS	3186 (0.48)	3393 (0.52)
No HS	9155 (0.45)	11305 (0.55)

	LVEF	
	< 50%	≥ 50%
HS	1075 (0.16)	5504 (0.84)
No HS	3789 (0.19)	**16671 (0.81)**

	MFR (in PET only)	
	< 2	≥ 2
HS	1606 (0.37)	2751 (0.63)
No HS	4449 (0.36)	**7934 (0.64)**

Frequency (%). Bold indicates sensitivity or specificity no less than 0.60.
Age of 60 years was used to define young (less than 60) and old (no less than 60), body mass index (BMI) of 30 kg/m^2 was used to define obesity (no less than 30 kg/m^2) and non-obesity (less than 30 kg/m^2), stress total perfusion deficit (TPD) of 5% was used to define abnormal (no less than 5%) and normal (less than 5%), and left ventricle ejection fraction (LVEF) of 50% was used to define abnormal (less than 50%) and normal (no less than 50%). HS – hepatic steatosis, CAC – coronary artery calcium, MFR – myocardial flow reserve, PET – positron emission tomography

Supplementary Figures

(A)

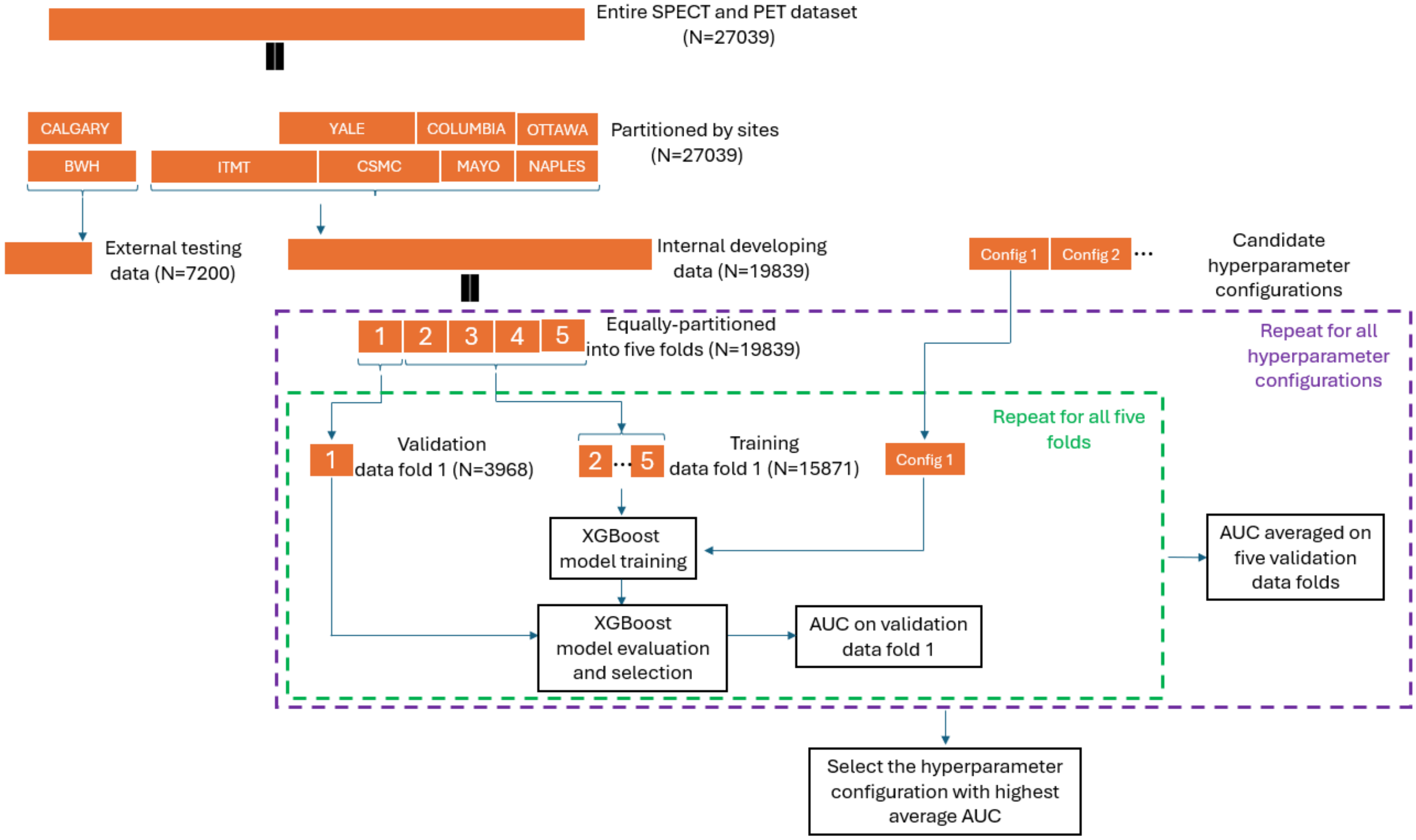

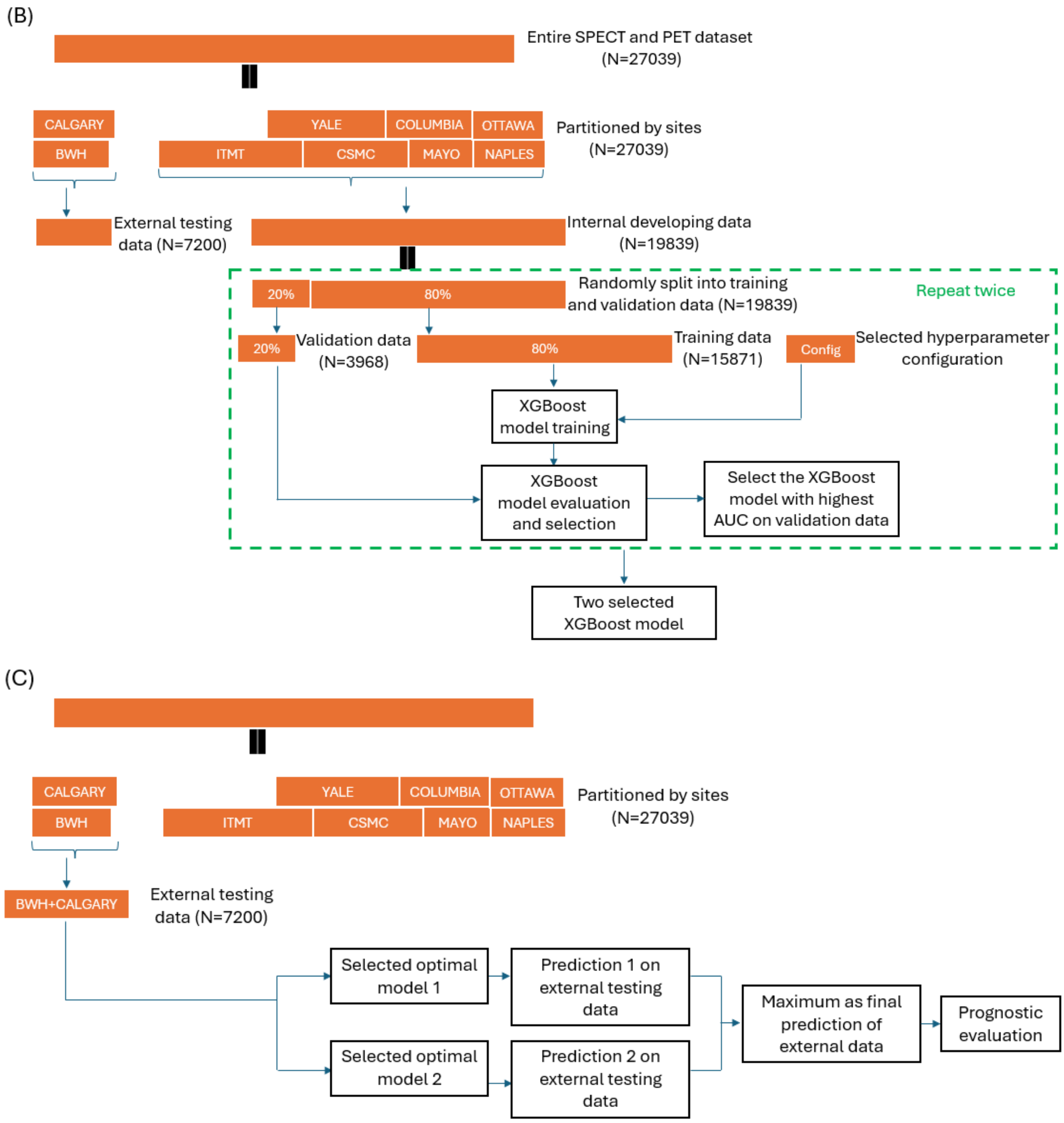

Supplementary Figure 1. eXtreme Gradient Boosting (XGBoost) model development and validation in external testing scheme where patient cases from PET site Brigham and Women's Hospital (BWH) and SPECT site University of Calgary were used as external site testing data while the cases from the other four PET sites and the other three SPECT sites were used for model development. (A): hyperparameter selection pipeline, (B): model training and selection pipeline, (C): model testing pipeline. PET – positron emission tomography, SPECT – single-photon emission computed tomography, BWH – Brigham and Women's Hospital, ITMT – Intermountain Healthcare, CSMC – Cedars-Sinai Medical Center, MYAO – Mayo Clinic, NAPLES – University of Naples Federico II, Yale – Yale University, CALGARY – University of Calgary, COLUMBIA – Columbia University, OTTAWA – University of Ottawa, AUC – area under receiver operating characteristic curve, XGBoost – eXtreme Gradient Boosting

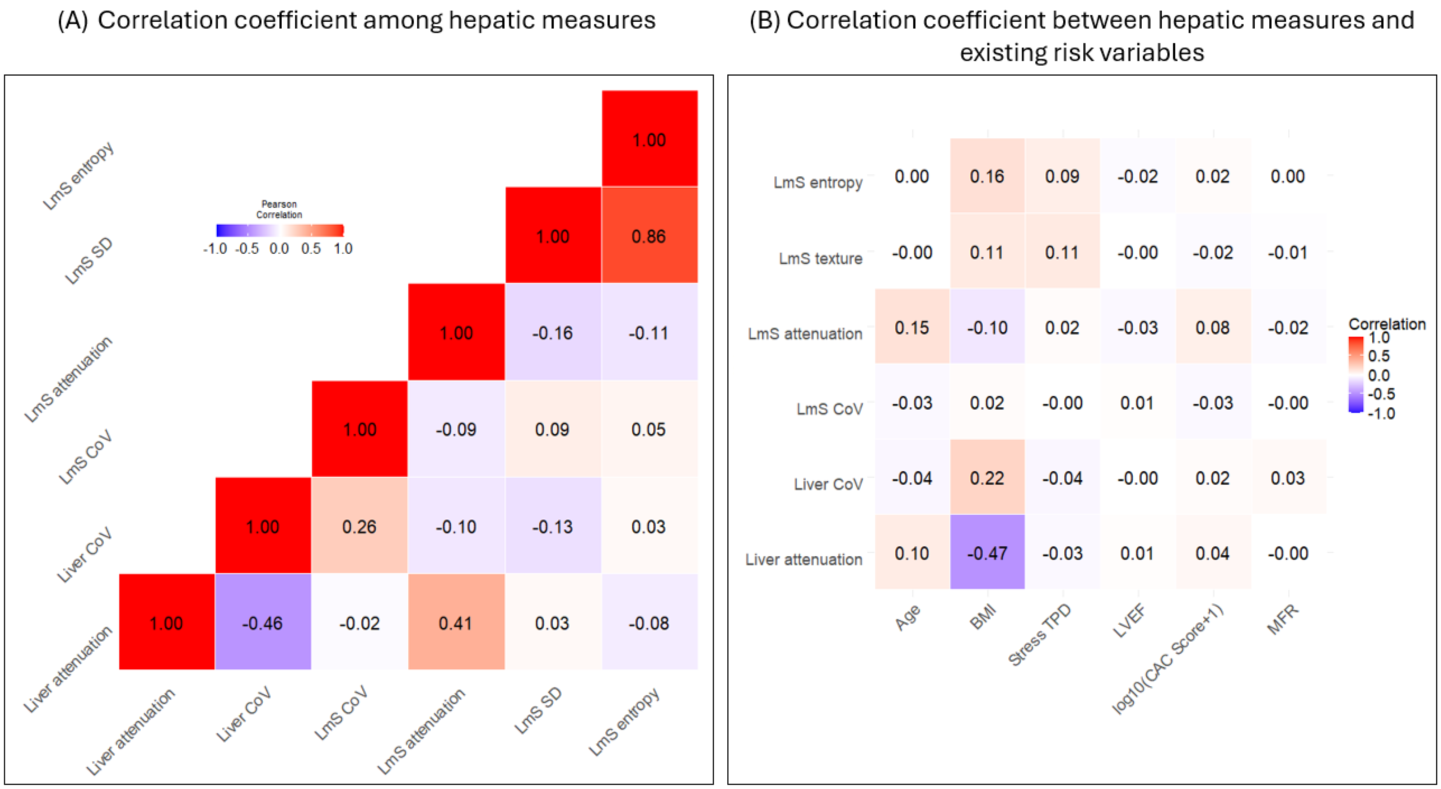

Supplementary Figure 2. Correlation plots of variables with VIF < 5. (A) among hepatic measures; (B) between hepatic measures and clinical/imaging variables. Correlation with MFR was only calculated for PET patients. Correlation coefficient for MFR was only calculated within PET patients. VIF – variance inflation factor, LmS – difference of liver minus spleen, SD – standard deviation, CoV – coefficient of variation, BMI – body mass index, TPD – total perfusion deficit, LVEF – left ventricular ejection fraction, CAC – coronary artery calcium, MFR – myocardial flow reserve.

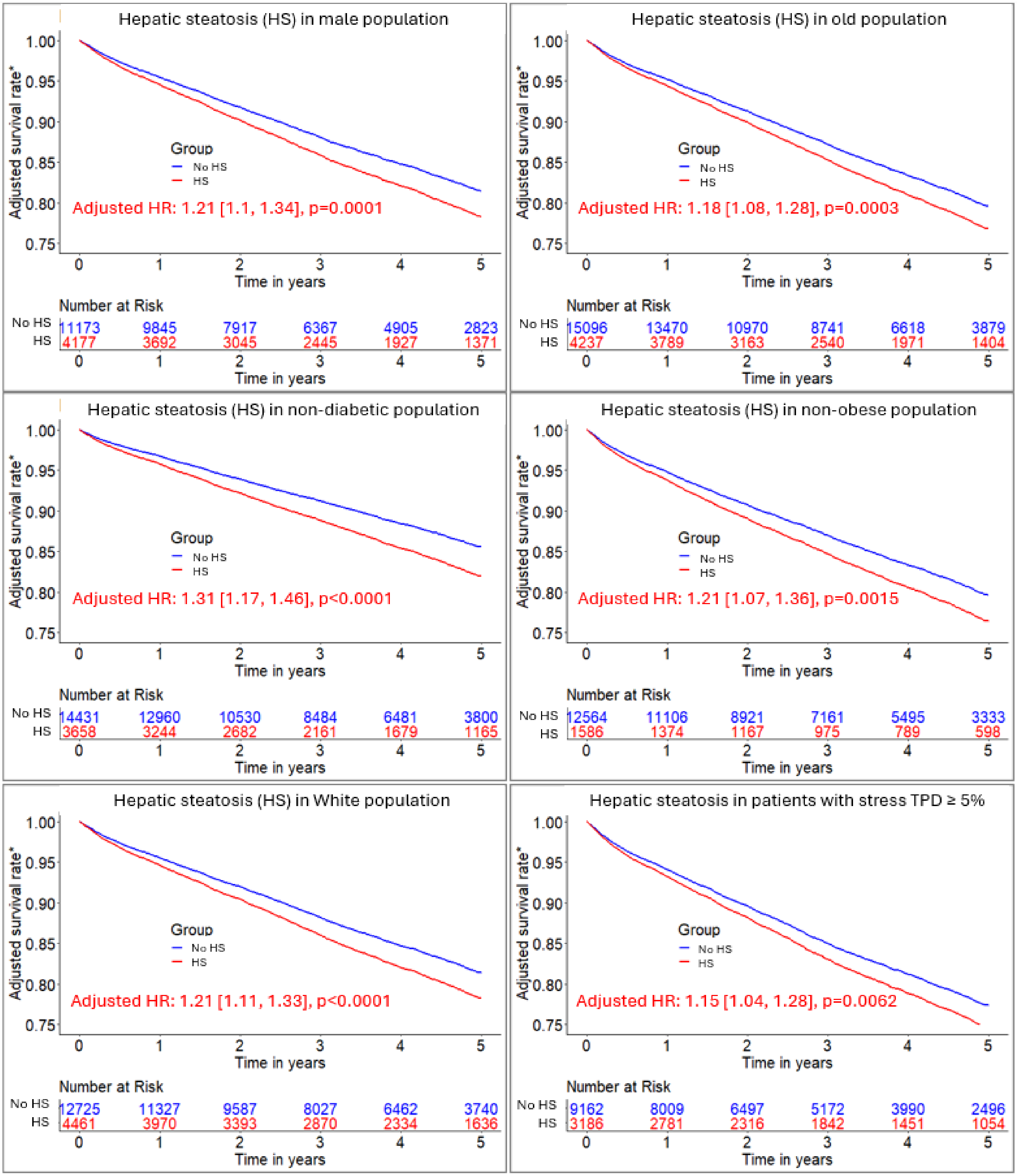

Supplementary Figure 3. Adjusted Kaplan-Meier curves of hepatic steatosis in male, old, non-obese, White, non-diabetic, and abnormal-perfusion populations, respectively from a cohort of 27039 patients. Patients without hepatic steatosis measures were

reference groups. Clinical and perfusion factors: sex, age, body mass index, hypertension, diabetes mellitus, dyslipidemia, family history of coronary artery disease, smoking, stress total perfusion deficit, left ventricle ejection fraction, and deep-learning-coronary artery calcium score. Patients were considered old if they had age ≥ 60 years, and non-obese if they had body mass index ≥ 30 kg/m^2. *Adjusted for clinical, perfusion factors and all the other hepatic quantifications. HR of age/BMI/log10(CAC score+1) was per unit increase. Female/LVEF ≥ 50%/Stress TPD<5%/low liver CoV/low LmS CoV/low LmS entropy/low LmS SD was reference group. For diabetes mellitus, dyslipidemia, family history of CAD, hypertension, and smoking, patients without them were the reference groups. Abnormal perfusion was defined as stress TPD ≥ 5%. BMI – body mass index, CAD – coronary artery disease, TPD – total perfusion deficit, LVEF – left ventricular ejection fraction, CAC – coronary artery calcium

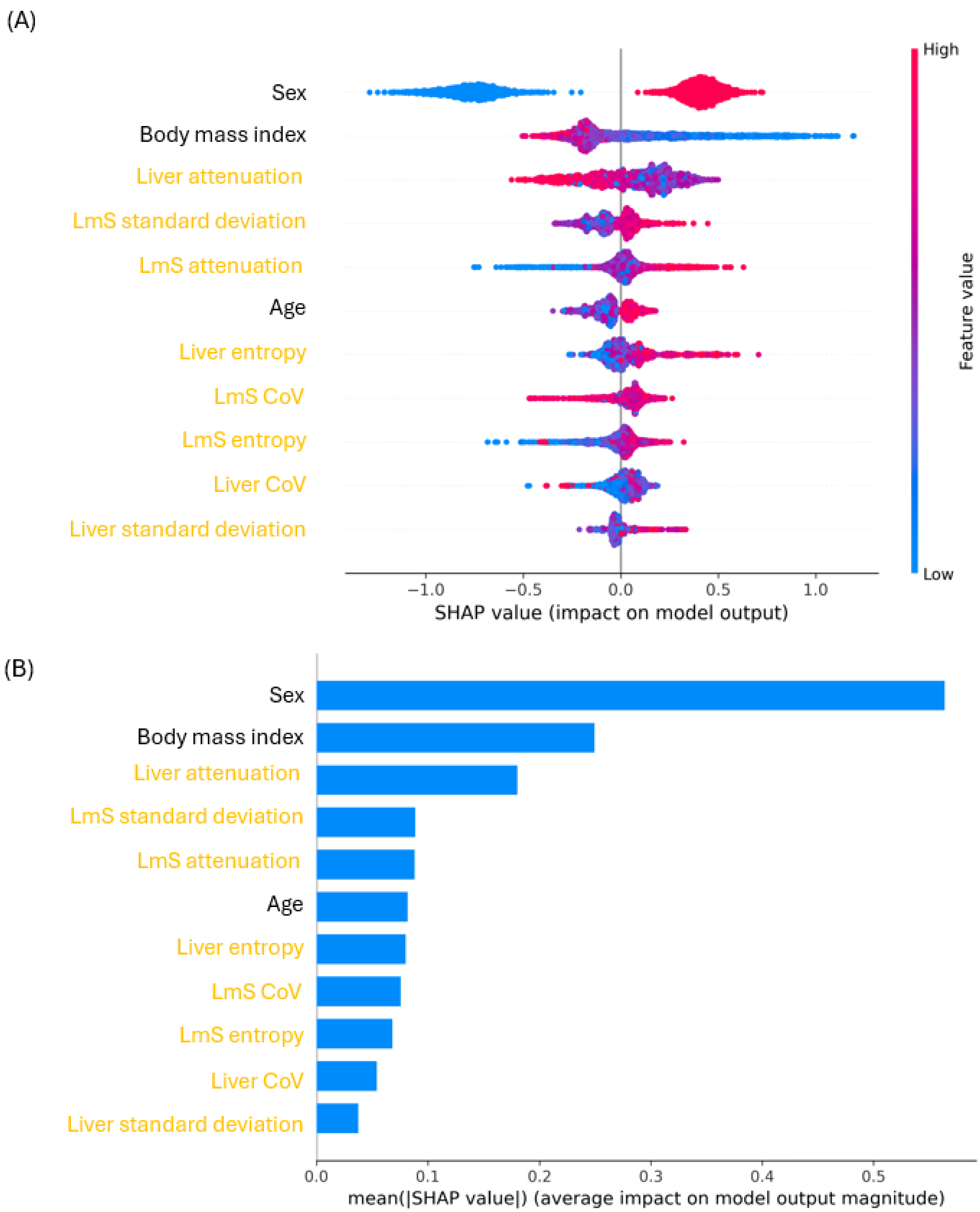

Supplementary Figure 4. Contribution of individual hepatic measure to liver risk index (LIRI). (A): scatter plot of SHAP value; (B): normalized mean absolute mean plot of SHAP value. Yellow indicates hepatic measures. Hepatic steatosis (HS) was determined according to the imaging criterion: a patient had HS if liver attenuation < 40 HU or liver minus spleen (LmS) attenuation < -10. LVEF

– left ventricular ejection fraction, CAC – coronary artery calcium, TPD – total perfusion deficit, CoV – coefficient of variation, CAD – coronary artery disease.

Reference

1. Otaki Y, Singh A, Kavanagh P, et al. Clinical Deployment of Explainable Artificial Intelligence of SPECT for Diagnosis of Coronary Artery Disease. *JACC Cardiovasc Imaging* 2022; 15: 1091-1102. 2021/07/19. DOI: 10.1016/j.jcmg.2021.04.030.
2. Lawrence DA, Oliva IB and Israel GM. Detection of hepatic steatosis on contrast-enhanced CT images: diagnostic accuracy of identification of areas of presumed focal fatty sparing. *AJR Am J Roentgenol* 2012; 199: 44-47. 2012/06/27. DOI: 10.2214/AJR.11.7838.
3. Wells MM, Li Z, Addeman B, et al. Computed Tomography Measurement of Hepatic Steatosis: Prevalence of Hepatic Steatosis in a Canadian Population. *Can J Gastroenterol Hepatol* 2016; 2016: 4930987. 2016/07/23. DOI: 10.1155/2016/4930987.
4. Hothorn T and Lausen B. On the exact distribution of maximally selected rank statistics. *Comput Stat Data An* 2003; 43: 121-137.
